# Development of Recoverable Magnetic Bimetallic ZIF-67 (Co/Cu) Adsorbent and Its Enhanced Selective Adsorption of Organic Dyes in Wastewater

**DOI:** 10.3390/molecules29204860

**Published:** 2024-10-14

**Authors:** Fuyan Zhang, Miaomiao Ma, Shuang Li, Yuting Zhou, Jian Zeng, Meiqi Huang, Qi Sun, Tao Le

**Affiliations:** 1 College of Life Sciences, Chongqing Normal University, Chongqing 401331, China; 2 Chongqing Key Laboratory of Conservation and Utilization of Freshwater Fishes, College of Life Sciences, Chongqing Normal University, Chongqing 401331, China

**Keywords:** magnetic adsorbent, bimetallic organic framework, selective adsorption, electrostatic interactions, water treatment

## Abstract

In the critical domain of wastewater treatment, the development of cost-effective, durable, and recyclable adsorbents with high adsorption capacities remains a significant challenge. This study introduces a novel magnetic bimetallic Metal–Organic Framework (MOF) adsorbent, MZIF-67-Co/Cu, doped with copper ions. The MZIF-67-Co/Cu adsorbent was successfully synthesized and structurally characterized, demonstrating remarkable selectivity for removing methyl orange (MO) from water. This high selectivity is attributed to the adsorbent’s high porosity and Lewis base properties at the coordinating metal ion center. The incorporation of Cu ions significantly enhances the porous architecture and increases the number of metal adsorption sites, leading to an impressive maximum MO adsorption capacity of 39.02 mg/g under optimized conditions (0.5 g/L adsorbent concentration, pH 3.0, 250 rpm agitation speed, adsorption time > 10 min). The adsorption kinetics closely follow the pseudo-second-order model, and the isotherm data fit well with the Langmuir model. The primary adsorption mechanisms involve electrostatic attraction and mesoporous interaction. This study highlights MZIF-67-Co/Cu as a highly efficient adsorbent with magnetic recovery capabilities, positioning it as a promising candidate for addressing critical issues in wastewater treatment.

## 1. Introduction

Widely utilized in industries like the textile, printing, plastic, and pharmaceutical industries, organic dyes present a substantial environmental hazard due to their enduring nature and potential harm. When released into water sources, they not only harm aquatic ecosystems, but also pollute drinking water reservoirs [1]. The escalating worry regarding environmental contamination from organic dyes necessitates the urgent development of efficient strategies to mitigate their negative effects. The adsorption techniques for wastewater treatment are considered to be environmentally friendly, low cost, chemically stable, easy to regenerate, and have good selectivity. A plethora of adsorbents have been applied to eliminate various organic dyes, including zeolites [2], polymers [3], calcium alginate [4], and carbon-based materials [5]. Among them, metal–organic frameworks (MOFs) have attracted significant attention in recent years due to their efficacy in removing pollutants from aqueous solutions [6].

MOFs consists of metal ions and organic ligands linked by coordination bonds. They offer benefits such as a large specific surface area, adjustable pore size, and numerous surface functional groups, making them promising for adsorption applications [6,7]. Researchers have successfully synthesized activated Fe-based MIL-88A as an adsorbent and utilized it for removal of various arsenic contaminants [8]. This study also investigated the adsorption behavior and mechanism of different arsenic contaminants on the activated MIL-88A. Nguyen et al. [9] synthesized MOF-808 using 1,3,5-benzenetricarboxylic acid as an organic ligand with metal zirconium ions. The adsorption of Zr-MOF on sunset yellow was 642 mg/g and on quinoline yellow was 731 mg/g. Due to weak interactions between MOFs and good dispersion in aqueous solutions, conventional MOFs are challenging to recycle in practical applications. This not only impacts their performance but also leads to potential secondary pollution. Furthermore, traditional MOFs suffer from slow adsorption rates and limited adsorption capacities when removing pollutants. Therefore, enhancing the adsorption capabilities of MOFs is crucial for improving their overall application performance.

Magnetic separation can offer a distinct advantage over conventional separation methods like centrifugation, filtration, and precipitation, as they can be easily separated under an external magnetic field, providing simpler operation and faster separation [10]. In recent years, magnetically functionalized MOFs have been surveyed using MOFs as adsorbents and Fe_3_O_4_ as the magnetic component for environmental applications. For instance, Qin et al. [11] designed and synthesized Fe_3_O_4_@UiO-66 with various combinatorial modes. Thanks to the porous structure of UiO-66 and the catalytic enhancement of Fe_3_O_4_, the nanocomposite material achieved efficient adsorption of aflatoxin B_1_. In recent years, the preparation of poly-MOFs by incorporating other transition metals has proven to be another effective method for enhancing their activity. In bimetallic MOFs, two inorganic metal nodes are utilized to combine two monometallic MOFs, resulting in significantly enhanced performance compared to their monometallic counterparts [12,13,14]. For example, the researchers devised and manufactured structurally stable Ni-Ti bimetallic MOF diffractions with highly dispersed metal species. The complex components and circular disk-like structure of these bimetallic MOF derivatives were found to significantly augment the dehydrogenation behavior of LiAlH_4_ [15]. In another study, researchers used a one-pot mixing ligand strategy to synthesize a magnetic multivariate MOF, named BTB/MOF-525@Fe_3_O_4_, for high-efficiency adsorption and rapid magnetic separation of bisphenol contaminants in the environment [16].

Consequently, based on the porous structure of ZIF-67 and the good catalytic activity of Cu-based MOF, we used a facile one-pot method to prepare a series of magnetic bimetallic MOFs, named MZIF-67-Co/Cu (x:1) (x = 0.5, 1, 2, 4, and 8). The adsorption properties and mechanisms of various dye molecules in MZIF-67-Co/Cu were thoroughly examined through material characterization and adsorption experiments using typical organic dyes as representative pollutants. The effects of the adsorption conditions and Co/Cu molar ratio on the selective separation of dyes from water were discussed. The development of magnetic bimetallic MOFs, particularly MZIF-67-Co/Cu, presents a novel approach to enhancing the adsorption capabilities for organic dyes in wastewater. By incorporating Cu ions into the ZIF-67 structure, we aim to optimize the porous architecture and increase the number of metal adsorption sites, thereby improving the adsorption efficiency. This study not only advances the field of magnetic bimetallic MOFs, but also contributes to the development of more effective and sustainable wastewater treatment technologies.

## 2. Results and Discussion

### 2.1. Characterizations of Synthesized Magnetic Bimetallic MOF Adsorbents

**Figure 1:**
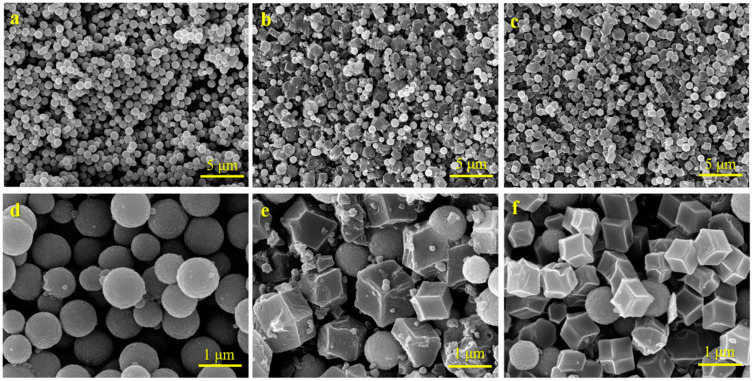
SEM observations of (**a**,**d**) Fe_3_O_4_, (**b**,**e**) Fe_3_O_4_/ZIF-67 (here denoted as MZIF-67), and (**c**,**f**) MZIF-67-Co/Cu (8:1) with low and high resolutions, respectively.

**Figure 2:**
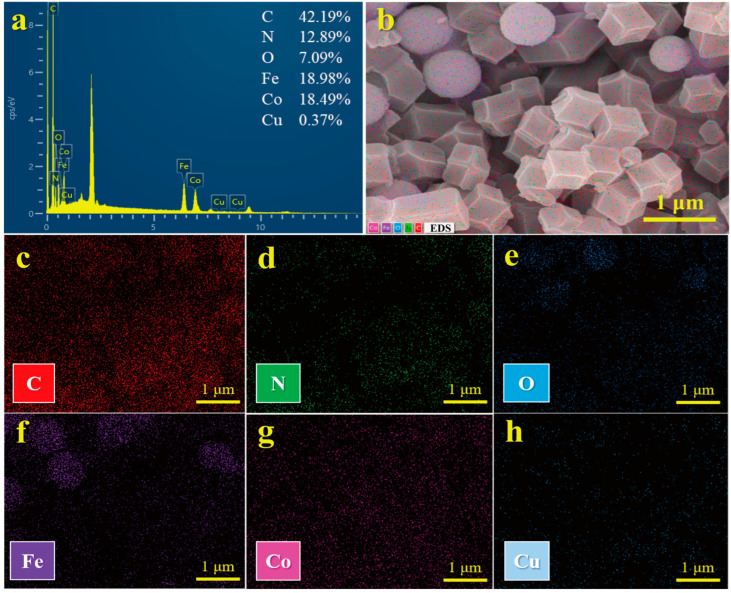
(**a**) Energy dispersive X-ray spectroscopy (EDS) spectrum and (**b**–**h**) elemental mappings (C, N, O, Fe, Co and Cu) of MZIF-67-Co/Cu (8:1).

SEM images (Figure 1a,d) clearly revealed that the synthesized Fe_3_O_4_ NPs were observed to be monodisperse and spherical in shape, with a mean diameter of 0.96 ± 0.12 nm. After combining Fe_3_O_4_ NPs with ZIF-67 (denoted as MZIF-67), SEM images showed that ZIF-67 had a typical rhombic dodecahedral structure with a uniform particle size of 500–700 nm (Figure 1b,e). Particularly, the Fe_3_O_4_ NPs grown on the ZIF-67 surface are much smaller than the pure Fe_3_O_4_ nanoparticles. When doped with copper, the magnetic bimetallic MZIF-67-Co/Cu (8:1) prepared were more uniform in particle size and had a smoother surface compared to MZIF-67 alone (Figure 1c,f). Furthermore, the Energy Dispersive X-ray Spectroscopy (EDS) spectrum (Figure 2a) and these EDS elemental mapping results indicate that the MZIF-67-Co/Cu (8:1) surface consists mainly of the elements C, N, O, Fe, and Co, with a small amount of Cu (Figure 2b–h). The above characterization results indicated the successful preparation of magnetic bimetallic MZIF-67-Co/Cu.

**Figure 3:**
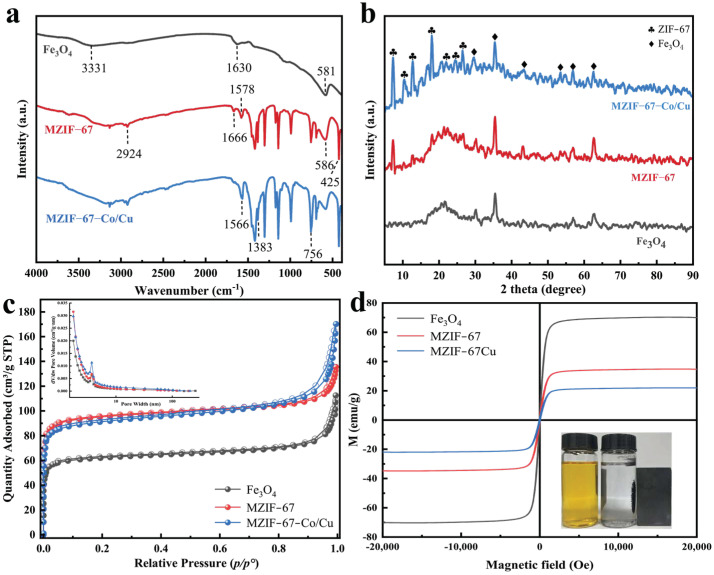
(**a**) FT-IR spectra, (**b**) XRD patterns, (**c**) nitrogen adsorption–desorption isotherms and pore-size distribution (inset), and (**d**) magnetic hysteresis curves of as-prepared Fe_3_O_4_, MZIF-67, and MZIF-67-Co/Cu, respectively.

The Fourier-transform infrared (FT-IR) spectra of Fe_3_O_4_ NPs, MZIF-67, and the magnetic bimetallic MZIF-67-Co/Cu exhibited characteristic absorption peaks in the range of 500–4000 cm^−1^ (Figure 3a). The peaks observed near 580–590 cm^−1^ for Fe_3_O_4_ NPs, MZIF-67, and MZIF-67-Co/Cu indicate characteristic vibrations of the Fe-O bonds, suggesting the formation of Fe_3_O_4_ NPs [17]. This further confirms the presence of the magnetic core, which is more pronounced in the bare magnetite nanoparticles [18]. For MZIF-67, the peak observed at 2924 cm^−1^ is associated with the stretching vibration of C-H in the aromatic ring of 2-methylimidazole. The absorption peak at 1666 cm^−1^ is associated with the stretching vibration of the C=N double bond, while the peak at 1578 cm^−1^ is associated with the stretching vibration of the N-H bond in the imidazole ring [19]. The band at 1350~1500 cm^−1^ corresponds to the tensile vibration of the imidazole ring, while the band at 900~1350 cm^−1^ represents the plane vibration of the imidazole ring. Additionally, the band at 500~800 cm^−1^ indicates the out-of-plane bending vibration of the imidazole ring, and the peak at 425 cm^−1^ is attributed to the tensile vibration of Co-N [20]. The MZIF-67-Co/Cu composite retains all the chemical bonds found in MZIF-67, demonstrating that it maintains the structural integrity of MZIF-67. The peaks observed in MZIF-67-Co/Cu at approximately 1566 cm^−1^ and 1383 cm^−1^ correspond to the coordination of Cu^2+^ and Co^2+^ with -OH groups, while the peak around 756 cm^−1^ represents the tensile and bending vibrations of Cu-O-H and Co-O-H bonds in MZIF-67-Co/Cu, aligning well with previous reports [21].

The crystal structures of the synthesized adsorbents were characterized using X-ray diffraction (XRD) (Figure 3b). The diffraction peaks of MZIF-67-Co/Cu at 18.2°, 29.6°, 35.4°, 43.3°, 53.5°, 56.9°, and 62.6° align closely with those of pure Fe_3_O_4_ NPs, which indicates a strong match between the two. These peaks correspond to the (111), (220), (311), (400), (422), (511), and (440) crystal planes [22,23], suggesting that the crystal structure of Fe_3_O_4_ in MZIF-67-Co/Cu remains largely unchanged. This finding is also consistent with the SEM results. Furthermore, the peaks of MZIF-67-Co/Cu at 7.3°, 10.4°, 12.7°, 18.0°, 22.1°, 24.6°, and 26.7° are in line with the characteristic peaks of ZIF-67 as reported in the literature [24], which are attributed to the (011), (002), (112), (222), (114), (233), and (134) crystal faces of ZIF-67. The main diffraction peaks of MZIF-67-Co/Cu are similar to those of ZIF-67. However, the characteristic diffraction peak of Cu is significantly lower, indicating that the introduction of Cu^2+^ replaces Co^2+^ in the lattice to a small extent. The results indicate that the addition of Cu^2+^ has a minimal effect on the crystal growth of the ZIF unit. Similar XRD results were found in Ce(III)-doped UiO-66. Yang et al. reported that the characteristic XRD peaks of Ce(III)-doped UiO-66 were in good agreement with those simulated based on UiO-66 single-crystal data, which suggested that the doped Ce(III) ions should be well-incorporated into the framework and partially substitute Zr(IV) ions in [Zr6O4(OH)4]^12+^ clusters [25].

The nitrogen adsorption–desorption isotherms of Fe_3_O_4_ NPs, MZIF-67, and MZIF-67-Co/Cu are depicted in Figure 3c, showcasing type-II adsorption isotherms with an inverted S shape [26]. The detected Brunauer–Emmett–Teller (BET) specific surface area of the two magnetic ZIF composites, MZIF-67 and MZIF-67-Co/Cu, increased significantly from 200.5 m^2^/g to 306.1 m^2^/g and 295.2 m^2^/g compared to pure Fe_3_O_4_ NPs. For the three adsorbents, their pore size distributions were calculated to be 3–5 nm (the inset of Figure 3c) by employing the Barret-Joyner-Halenda (BJH) method, which confirms the mesoporous structure [27].

The above characterization results indicate that metal doping can introduce variability into the coordination environment of organic ligands and central metal ions, resulting from the competitive binding of diverse metal cations to the ligands [28]. Importantly, while doping may not necessarily alter the geometric surface area, it typically increases the chemically active surface area. Consequently, the superior performance of metal-doped MOFs compared to their undoped counterparts and other potential adsorbents can be attributed to a synergistic effect of heightened structural heterogeneity and an increased abundance of chemical adsorption sites [29]. These factors collectively contribute to an expansion of the active or specific surface area available for adsorption. Furthermore, doping can improve reactivity by modulating the reaction energy landscape, potentially rendering it more favorable and/or lowering the energy barriers associated with rate-determining steps [30].

Magnetic hysteresis curves are commonly used to analyze the magnetic properties of different materials. In Figure 3d, the hysteresis curves of Fe_3_O_4_, MZIF-67, and MZIF-67-Co/Cu are presented. These hysteresis curves exhibit an S-shaped pattern with superparamagnetic behavior. The saturation magnetization of Fe_3_O_4_ (70.3 emu/g), MZIF-67 (34.8 emu/g), and MZIF-67-Co/Cu (22.0 emu/g) decreases sequentially, suggesting successful attachment of non-magnetic ZIF-67 or ZIF-67-Co/Cu to Fe_3_O_4_ NPs. This connection leads to a reduction in saturation magnetization, while still allowing rapid magnetic separation of MZIF-67-Co/Cu under the influence of an external magnetic field as shown in the inset of Figure 3d.

### 2.2. Selective Adsorption of Organic Dyes on Magnetic Bimetallic MOF Adsorbents

**Figure 4:**
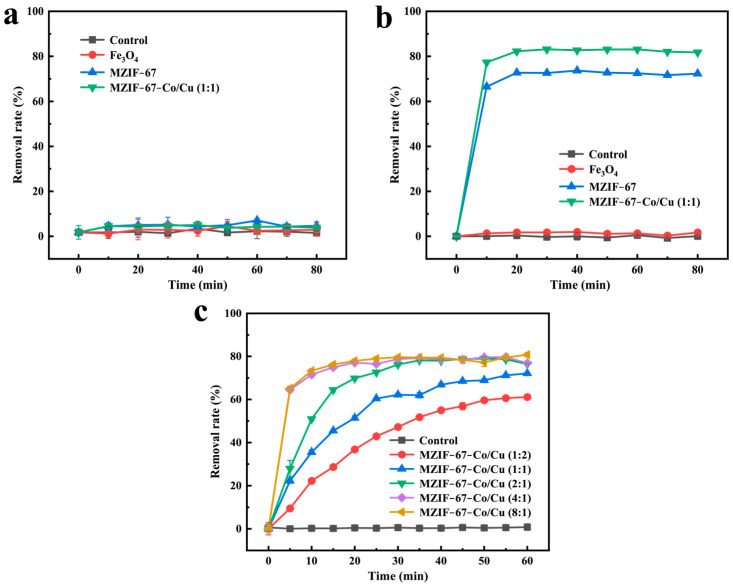
Selective adsorption of typical (**a**) cationic dyes MB and (**b**) anionic dyes MO by different adsorbents, including the control, Fe_3_O_4_ NPs, MZIF-67, and MZIF-67-Co/Cu. (**c**) Effect of different Co and Cu ion doping molar ratios on the adsorption of MO dyes by MZIF-67-Co/Cu.

Herein, the adsorption experiments of different adsorbents to MB (cationic dye) and MO (anionic dye) were performed, due to their similar molecular structures and sizes. The Fe_3_O_4_ NPs lack the ability to adsorb and remove both dyes, as evidenced by the results presented in Figure 4a,b. The magnetic MOF adsorbents, MZIF-67 and MZIF-67-Co/Cu, showed selective adsorption behavior for the two dyes studied, as evidenced by almost no adsorption properties for MB (Figure 4a), whereas adsorption equilibrium was reached after 20 min for MO (Figure 4b). The significant difference in the adsorption capacity of a positively charged adsorbent for methyl orange (MO) versus methylene blue (MB) can be attributed to the electrostatic interactions between the adsorbent and the dyes [31]. Specifically, MO is an anionic dye with a negatively charged sulfonate group, which is attracted to the positively charged surface of the adsorbent. In contrast, MB is a cationic dye with a positively charged dimethylamino group, which repels the positively charged adsorbent surface, resulting in negligible adsorption.

In addition, the adsorptive removal of MO by bimetallic MZIF-67-Co/Cu was 1.2 times higher than that of the monometallic MZIF-67. Bimetallic MOF exhibits superior adsorption capabilities compared to monometallic MOF due to the presence of copper ions in the crystal lattice of ZIF-67. These copper ions provide more unsaturated metal sites, providing valence electrons that enhance the interaction between the dye and the metal center, ultimately increasing the adsorption capacity [32]. The impact of varying Co and Cu molar doping ratios on the adsorption of MO by magnetic bimetallic MOF adsorbents was then investigated. It was observed that, as the Co and Cu doping molar ratio increased from 0.5:1 to 8:1, the adsorption capacity and removal efficiency of MZIF-67-Co/Cu for MO dyes increased significantly (Figure 4c). The result indicates that the optimal adsorption performance and removal efficiency for MO dyes using MZIF-67-Co/Cu as the magnetic adsorbent is achieved when the Co and Cu doping molar ratio exceeds 4:1. This can be attributed to the significant role of Co ions in bimetallic MOFs, where an increase in the Co(NO_3_)_2_ ratio results in a more refined material with enhanced MO adsorption capacity [33]. Additionally, the 8:1 ratio provided a larger margin of error in case of slight variations in synthesis conditions. We understand that using the 4:1 ratio could potentially reduce Co consumption. However, given the similarities in performance, we chose MZIF-67-Co/Cu at a Co and Cu molar ratio of 8:1 for MO adsorption in subsequent experiments.

### 2.3. Optimization of the Magnetic Adsorption Conditions

**Figure 5:**
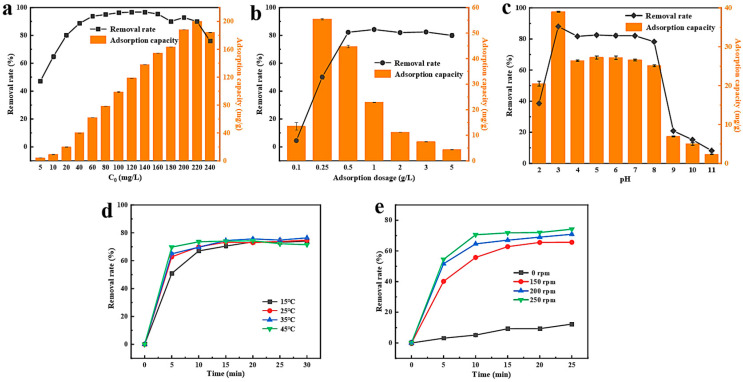
Effects of (**a**) initial MO concentration, (**b**) adsorbent dosage, (**c**) solution pH, (**d**) adsorption temperature, and (**e**) agitation speed on adsorption efficiency of the magnetic bimetallic MZIF-67-Co/Cu adsorbent.

Due to the significance of reaction conditions for dye adsorption, we conducted a thorough optimization of reaction parameters. As the initial concentration of MO dye increases, the adsorption removal rate of MO by MZIF-67-Co/Cu initially rises, reaches equilibrium, and then declines (Figure 5a). Specifically, at an initial concentration of 140 mg/L, the adsorption removal rate of MO by MZIF-67-Co/Cu peaks at 96.80%. At a dye concentration of 220 mg/L, the maximum adsorption capacity of MZIF-67-Co/Cu for MO dyes was measured at 200.1 mg/g. This trend could be due to the constant number of adsorption sites available on the MZIF-67-Co/Cu. At lower dye concentrations, more MO molecules bind to these sites, leading to increased removal rates and adsorption capacity [34]. Conversely, at higher dye concentrations, the limited adsorption sites result in decreased removal rates and adsorption capacity. Based on these findings, an initial concentration of MO dyes at 140 mg/L was selected for subsequent condition optimization in this study. If higher concentrations of MO are encountered in the actual system, the purpose of removing MO from the water can be achieved by increasing the adsorbent content or diluting the contaminated water.

The adsorption of MO dye by MZIF-67-Co/Cu increases and then decreases with increasing adsorbent concentration (Figure 5b). The maximum adsorption capacity for MO dye is reached when the concentration of MZIF-67-Co/Cu is 0.25 g/L. This phenomenon may be attributed to the simultaneous occupation of limited MO molecules by active sites on the adsorbent, resulting in the formation of unsaturated adsorption sites on the surface of MZIF-67-Co/Cu [35]. When the adsorbent concentration exceeds 0.5 g/L, the adsorption removal rate of MO dyes by MZIF-67-Co/Cu remains almost unchanged, indicating that mass transfer equilibrium between MO molecules and the adsorbent in the solid and liquid phases has been achieved. Taking all factors into account, the selected concentration of MZIF-67-Co/Cu is 0.5 g/L. The influence of solution pH on the adsorption performance of MZIF-67-Co/Cu for MO removal is presented in Figure 5c. The adsorption capacity and removal rate of MO dyes by MZIF-67-Co/Cu initially increase with the solution pH, reach a peak at pH = 3 with an adsorption capacity of 39.1 mg/g and a removal rate of 88.03%, and then decrease. This observation can be attributed to the proximity of the MOF material’s isoelectric point to pH = 3, indicating that the adsorption of MO by MZIF-67-Co/Cu is predominantly governed by electrostatic interactions between dye molecules and the adsorbent surface. The study also examined how reaction temperature and shaker speed affect the adsorption performance of MZIF-67-Co/Cu for MO removal. The results show that reaction temperature has minimal impact on the removal efficiency, but higher temperatures lead to faster equilibrium attainment in the adsorption reaction (Figure 5d). Additionally, increasing agitation speeds result in more frequent and intense contact between MZIF-67-Cu and MO, significantly enhancing the adsorption effectiveness of the adsorbent on dye molecules (Figure 5e).

### 2.4. Adsorption Kinetics and Isotherms

**Figure 6:**
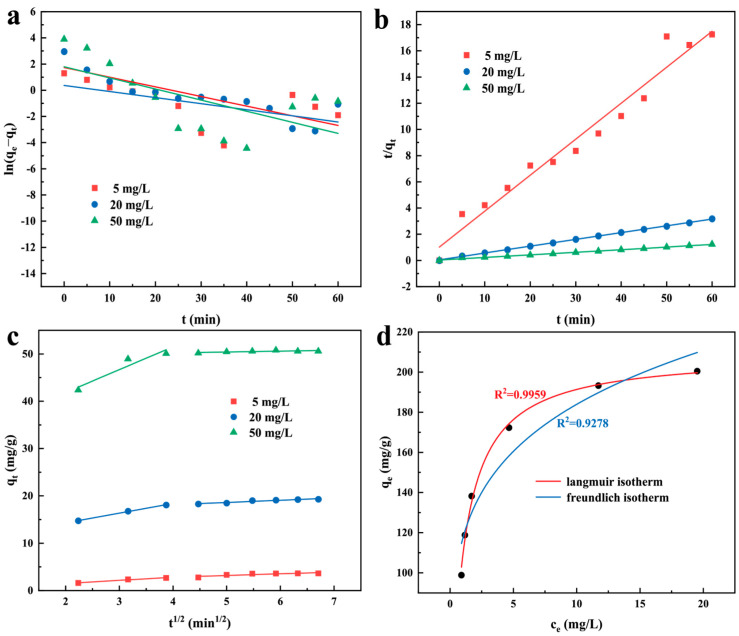
(**a**) Pseudo-first-order kinetics, (**b**) pseudo-second-order kinetics, (**c**) intraparticle diffusion kinetics and (**d**) equilibrium isotherms of MO adsorption on the magnetic bimetallic MZIF-67-Co/Cu adsorbent.

The adsorption process of MO by MZIF-67-Co/Cu was analyzed using both pseudo-first-order and pseudo-second-order models. The adsorption kinetics and fitting parameters are shown in Figure 6a,b. The R^2^ values of the pseudo-second-order kinetic model are higher than those of the pseudo-first-order kinetic model, as shown in Table 1. Furthermore, the calculated equilibrium adsorption capacity (q_e,cal_) closely aligned with the experimental equilibrium adsorption capacity (q_e,exp_). These findings suggest that the adsorption process of MZIF-67-Co/Cu for MO removal adhered more closely to the pseudo-second-order kinetic model and is likely driven by chemisorption or chemical adsorption [36]. Furthermore, as the concentrations of MO increased from 5 to 50 mg/L, the k_2_ values of the pseudo-second-order model displayed a clear decline from 0.0742 to 0.0116. This suggests that enhancing the initial MO concentration can significantly boost the driving force between the magnetic adsorbent and dye molecules, which was also observed in Figure 5a.

Furthermore, the Weber-Morris model was applied to corroborate whether the adsorption process is governed by external diffusion, internal diffusion, or a combination of both. Figure 6c illustrates plots of q_t_ versus t^1/2^ linear regression analysis of q_t_ versus t^1/2^, with corresponding kinetic parameters detailed in Table 2. The adsorption process of MZIF-67-Co/Cu for MO removal can be segmented into two distinct stages. Initially, the steeper slope is attributed to the boundary layer effect [37], primarily involving the transportation of dye molecules from the aqueous solution to the adsorbent surface. Subsequently, the second stage showcases intra-particle diffusion of MO molecules across the abundant active sites of the magnetic bimetallic MZIF-67-Co/Cu adsorbent [38]. The obtained value for k_1_ exhibited a notable increase compared to k_2_, implying that the transfer of mass occurred at a faster rate in comparison to the diffusion within the particle. The findings suggested that the adsorption process of the MZIF-67-Co/Cu could potentially be influenced by both external mass transfer and intraparticle diffusion [39]. Moreover, intraparticle diffusion was not the rate-limiting step as indicated by the non-zero C values [40].

In order to analyze the adsorption behavior of MO on the magnetic bimetallic MZIF-67-Co/Cu, the commonly used Langmuir and Freundlich isotherm models were employed to study the experimental equilibrium adsorption results. The isotherm models (Langmuir and Freundlich) can be seen in Figure 6d, with the parameters obtained from the fitting process listed in Table 3. Both models showed a good fit to the experimental data (R^2^ > 0.92). Compared to the Freundlich model (R^2^ = 0.9278), the Langmuir model exhibited a higher R^2^ value (R^2^ = 0.9959) and is closer to 1. The results showed that the adsorption process was closer to the Langmuir isotherm model than the Freundlich isotherm model, indicating that the adsorption of MO by MZIF-67-Co/Cu was mainly based on monolayer adsorption [41]. Hence, the adsorption mechanism of the magnetic bimetallic MZIF-67-Co/Cu in relation to MO involves a multifaceted physicochemical process that may involve charge-transfer-mediated chemical reactions. The high adsorption capacity of the prepared MOF adsorbent can be attributed to various synergistic factors including its unique surface architecture and micropore volume, the electrostatic interactions between the dye molecules and the adsorbent, as well as the presence of functional groups on the surface of the MOF adsorbent.

## 3. Materials and Methods

### 3.1. Chemicals and Materials

Ferric chloride hexahydrate (FeCl_3_·6H_2_O), ethylene glycol, anhydrous sodium acetate, 2-methylimidazole (2-MI), cobalt nitrate hexahydrate [Co(NO_3_)_2_·6H_2_O], copper nitrate [Cu(NO_3_)_2_], methanol (CH_3_OH), methylene blue (MB), and methyl orange (MO) were obtained from Aladdin Co., Ltd. (Shanghai, China) and were all analytical-grade. Ultra-pure water (18.25 MΩ/cm) was used throughout all experiments via a Milli-Q system (Millipore, Bedford, MA, USA).

### 3.2. Synthesis of the Magnetic Bimetallic MOF Adsorbents

#### 3.2.1. Preparation of Fe_3_O_4_ Nanoparticles

The magnetic carrier Fe_3_O_4_ nanoparticles (denoted as Fe_3_O_4_ NPs) were first prepared by a hydrothermal process [42]. In a typical synthesis procedure, 2.7 g of FeCl_3_·6H_2_O was dissolved in 50 mL of ethylene glycol and stirred vigorously for 30 min to give a yellow transparent solution. Following this, 5.75 g of anhydrous sodium acetate was added to the solution and stirred for another 30 min, resulting in a yellow turbid liquid. The mixture was transferred to a Teflon-lined stainless-steel autoclave and heated at 200 °C for 8 h. The resulting Fe_3_O_4_ NPs were washed repeatedly with ethanol and water until reaching a neutral pH, then vacuum dried at 60 °C for 12 h.

#### 3.2.2. Preparation of the Magnetic ZIF-67

The magnetic ZIF-67 (MZIF-67) adsorbents were synthesized following a previously reported method [43]. Fe_3_O_4_ NPs (0.23 g, 1 mmol), 2-MI (3.94 g, 48 mmol), and Co(NO_3_)_2_·6H_2_O (1.75 g, 6 mmol) were individually dissolved in 50 mL of methanol to obtain solutions A, B, and C. Solution B was then added to solution A and stirred continuously at room temperature. After a 1 h reaction, solution C was introduced to the mixture and allowed to react for an additional 24 h. The resulting MZIF-67 adsorbents were collected via magnetic separation, washed with methanol, and dried under vacuum at 60 °C for 10 h.

#### 3.2.3. Preparation of the Magnetic Bimetallic ZIF-67

The preparation method of the magnetic bimetallic ZIF-67 (MZIF-67-Co/Cu) is similar to that of MZIF-67, with the only difference being the replacement of 6 mmol of Co(NO_3_)_2_·6H_2_O with varying proportions of Co(NO_3_)_2_·6H_2_O and Cu(NO_3_)_2_ at ratios of 1:2, 1:1, 2:1, 4:1, and 8:1, respectively.

### 3.3. Characterizations of the Magnetic Adsorbents

The structural morphologies of as-prepared magnetic adsorbents were analyzed by a Quantum FEG 650 scanning electron microscope (SEM). Fourier transform infrared spectrometer (FTIR) and X-ray diffraction (XRD) spectrum of magnetic adsorbents were recorded via IFS120HR Fourier transform infrared spectroscopy (FEI, Hillsboro, OR, USA) and Rigaku D/Max-2400 X-ray diffractometer (Shimadzu, Rigaku, Japan), respectively. Hysteresis loops of magnetic materials were measured using a PPMS type vibrating sample magnetometer (VSM) with a magnetic field range of −20,000 to 20,000 Oe and a temperature of 25 °C. The specific surface areas of the adsorbents were determined by Brunauer-Emmett-Taylor (BET) analysis, using an ASAP2020 automatic gas adsorption analyzer (Thermo Fisher Company, Arbor, MI, USA).

### 3.4. Batch Adsorption Experiments

In order to evaluate the adsorption capacity of as-prepared magnetic bimetallic MOF adsorbents, two hydrophilic dyes, MB and MO, were used as model dyes for the adsorption experiments. In a typical adsorption procedure, different magnetic materials, including Fe_3_O_4_ NPs, MZIF-67, and MZIF-67-Co/Cu, were each weighed at 50 mg and added to a 50 mL dye aqueous solution with an initial concentration of 20 mg/L. Dark adsorption took place at room temperature, with a blank control group set up simultaneously. The magnetic material with the most effective adsorption was chosen as the adsorbent, and the impacts of initial dye concentrations (5~240 mg/L), adsorbent dosages (0.1~5 g/L), solution pH (2~11), temperature (15~45 °C), and agitation speed (0~250 rpm) on adsorption removal of dyes were studied. Following magnetic separation of the adsorbent, changes in dye concentration pre- and post-adsorption were measured using UV-visible spectrophotometry. Subsequently, adsorption efficiency and capacity were calculated according to Equations (1) and (2) [44], as follows:

(1) $$\mathrm{Removal}\;\mathrm{rate}\;(\%)=\frac{C_{0}-C_{t}}{C_{0}}\;\times 100\%$$

(2) $$\mathrm{Adsorption}\;\mathrm{capacity}\;(\mathrm{mg}/g)=\frac{\left(C_{0}-C_{t}\right)\times V}{m}$$

where *C*_0_ (mg/L) is the initial concentration of dye; *C_t_* (mg/L) is the remaining dye concentration at time *t*. *V* (L) is the volume of reaction liquid; *m* (g) is the dosage of adsorbent.

### 3.5. Adsorption Kinetic and Adsorption Isotherm Studies

To comprehensively analyze the kinetic adsorption process of dyes by the magnetic adsorbents, we utilized various kinetic models, including pseudo-first-order and pseudo-second-order, as well as the intraparticle diffusion model. To investigate the adsorption isotherm, we employed the Langmuir and Freundlich isotherm models to fit the experimental data. The details of these models refer to Equations (3)–(7), respectively [44]:

ln(Q_e_ − Q_t_) = −k_1_t + lnQ_e_ (3)

(4) $$\frac{t}{Q_{t}}=\frac{t}{Q_{e}}+\frac{1}{k_{2}Q_{e}^{2}}$$

Q_t_ = k_p_t^0.5^ + C (5)

where Q_t_ (mg/g) is the adsorption capacity of dye at time *t*; k_1_ (min^−1^) is the adsorption rate constant of pseudo-first-order kinetic; *k*_2_ [g/(mg·min)] is the adsorption rate constant of pseudo-second-order kinetic; k_p_ [mg/(g·min^1/2^)] is the rate constant of intraparticle diffusion; C (mg/g) is the model constant of intraparticle diffusion.

(6) $$\frac{C_{e}}{Q_{e}}=\frac{C_{e}}{Q_{m}}+\frac{1}{Q_{m}K_{L}}$$

(7) $${\mathrm{lnQ}}_{e}={\mathrm{lnK}}_{F}+\frac{1}{n}{\mathrm{lnC}}_{e}$$

where *C_e_* (mg/L) is the concentration of dye in equilibrium; *Q_e_* and *Q_m_* (mg/g) are, respectively, the adsorption capacity and maximum adsorption capacity of dye at equilibrium; *K_L_* (L/mg) is the Langmuir adsorption equilibrium constant; *K_F_* (mg/g) is the Freundlich adsorption equilibrium constant; 1/*n* is the Freundlich adsorption strength constant.

### 3.6. Statistical Analysis

All experiments were conducted in triplicate, and the results are presented as means ± standard deviations. A Tukey’s test was employed to identify significant differences in the results of the one-way analysis of variance (ANOVA). The data were analyzed using the statistical software package SPSS version 22.0 (SPSS Inc., Chicago, IL, USA).

## 4. Conclusions

A series of magnetic bimetallic Metal–Organic Framework (MOF) adsorbents, specifically MZIF-67-Co/Cu with varying Co and Cu molar doping ratios, were successfully synthesized via a straightforward solvothermal method. These adsorbents exhibited remarkable efficiency in selectively removing anionic organic dyes such as methyl orange (MO), leveraging the unique electrostatic interactions. A thorough investigation revealed that the incorporation of Cu ions significantly enhanced the porous architecture of MZIF-67, introducing additional metal adsorption sites and boosting its MO adsorption capacity. The study underscored the critical impact of factors like MO concentration, adsorbent dosage, solution pH, and agitation speed on the adsorbent performance, while temperature played a minor role in influencing the equilibrium rate of the adsorption reaction. Notably, the adsorption data aligned well with both the pseudo-second-order kinetic model and the Langmuir isotherm model, confirming the chemisorptive nature of the process and its monolayer molecular adsorption characteristics. The rapid magnetic separation capability of MZIF-67-Co/Cu underscores its immense potential for application in wastewater treatment, representing a significant advancement in the field.

## Figures and Tables

**Table 1:** Kinetic model parameters of MO adsorption on MZIF-67-Co/Cu.

C_0_ (mg/L)	q_e_, exp (mg/g)	Pseudo-First-Order-Kinetics			Pseudo-Second-Order-Kinetics		
		q_e_, cal (mg/g)	k_1_ (min^−1^)	R^2^	q_e_, cal (mg/g)	k_2_ (g min^−1^ min^−1^)	R^2^
5	3.63	1.45	0.05	0.3208	3.64	0.07	0.9643
20	19.15	5.65	0.07	0.7784	19.30	0.06	0.9995
50	49.19	6.00	0.08	0.3878	50.76	0.01	0.9952

**Table 2:** Intraparticle diffusion model parameters of MO adsorption on the MZIF-67-Co/Cu.

C_0_ (mg/L)	First Stage			Second Stage		
	k_1_ (mg g^−1^h^−1/2^)	c_1_ (mg/g)	R^2^	k_2_ (mg g^−1^h^−1/2^)	c_2_ (mg/g)	R^2^
5	0.65	0.20	0.9734	0.35	1.43	0.7217
20	2.04	10.22	0.9973	0.47	16.25	0.9187
50	4.84	32.17	0.9060	0.18	49.49	0.5238

**Table 3:** Isotherm parameters of MO adsorption on the MZIF-67-Co/Cu.

Langmuir			Freundlich		
K_L_ (L/mg)	q_m_ (mg/g)	R^2^	K_F_	n	R^2^
1.07	209.22	0.9959	116.92	5.08	0.9278

## Data Availability

Data are contained within the article.
